# Artesunate Alleviates Inflammatory Injury in Neonatal Acute Respiratory Distress Syndrome Through CCL5‐Mediated Pyroptosis of M2 Macrophage Subsets

**DOI:** 10.1155/jimr/7437447

**Published:** 2026-08-05

**Authors:** Hai-Ran Ma, Yan-Mei Xie, Yu-Xuan Zhang, Chong-He Cui, Jing Liu

**Affiliations:** ^1^ Department of Paediatrics, The Second School of Clinical Medicine, Southern Medical University, Guangzhou 510280, China, fimmu.com; ^2^ Department of Neonatology and NICU, Beijing Obstetrics and Gynecology Hospital, Capital Medical University, Beijing Maternal and Child Health Care Hospital, Beijing 100026, China, ccmu.edu.cn; ^3^ Department of Neonatology and Neonatal Intensive Care Unit, Huizhou Central People’s Hospital, Huizhou 516008, China; ^4^ Department of Paediatrics, The Seventh Medical Center of PLA General Hospital, Beijing 100010, China; ^5^ Division of Orthopedics and Traumatology, Department of Orthopedics, Nanfang Hospital, Southern Medical University, Guangzhou 510515, Guangdong Province, China, fimmu.com

**Keywords:** artesunate, CCL5, M2 macrophage, NARDS, pyroptosis

## Abstract

Neonatal acute respiratory distress syndrome (NARDS) is a life‐threatening respiratory disorder characterized by high mortality and significant long‐term morbidity. Increasing evidence suggests that pyroptosis contributes to pulmonary tissue injury in ARDS. In this study, single‐cell RNA sequencing (scRNA‐seq) was utilized to investigate the molecular mechanisms underlying pyroptosis within the NARDS microenvironment. Our analysis identified chemokine (C–C motif) ligand (CCL5) expression in M2 macrophages as a potential regulator of pyroptosis associated with impaired tissue repair. Mechanistically, CCL5 was linked to the activation of the pyroptotic pathway through interactions with inflammasome components, promoting caspase‐11‐dependent gasdermin D (GSDMD) cleavage and interleukin‐1β (IL‐1β) release. Network pharmacology analysis identified artesunate, a clinically approved antimalarial drug, as a potential inhibitor of CCL5 expression in patients with NARDS. Functional validation showed that artesunate attenuated M2 macrophage pyroptosis, reduced pulmonary injury, and promoted tissue repair. Collectively, our findings identify CCL5 as a potential regulator of macrophage pyroptosis and support the therapeutic potential of artesunate for modulating pyroptosis‐associated inflammation in NARDS. This study provides a mechanistic framework for the development of CCL5‐targeted interventions and highlights the dual anti‐inflammatory and pro‐repair properties of artesunate in NARDS treatment.

## 1. Introduction

Neonatal acute respiratory distress syndrome (NARDS) is a severe respiratory disorder characterized by hypoxemia, inflammatory exudation, and reduced pulmonary compliance [1, 2]. The disease progresses rapidly and is associated with high morbidity and mortality, posing a severe threat to neonatal survival [3]. With a mortality rate as high as 30%–40%, NARDS typically presents with cyanosis and dyspnea within 48 h of onset [4]. Without timely intervention, it may progress to respiratory failure and remains a major cause of neonatal mortality [5, 6]. Current management focuses on treating the underlying cause and correcting hypoxemia to improve lung expansion, enhance blood oxygenation, and restore pulmonary compliance [7]. However, diagnostic and therapeutic outcomes remain suboptimal because of the unique physiological characteristics of neonates [8]. Consequently, a better understanding of the pathogenic mechanisms and molecular features of NARDS is needed to develop targeted therapies and improve clinical outcomes [9, 10].

Several studies have shown that cell death plays an important role in the development and occurrence of lung injury in NARDS [11–13]. Recent studies suggest that under different pathological conditions, pyroptosis, a novel form of programmed cell death closely related to inflammation, can trigger an inflammatory response in NARDS and exacerbate lung tissue damage [14]. These inflammatory mediators cause alveolar damage and pulmonary edema, promote lung tissue damage, and ultimately aggravate lung function deficits [15]. Therefore, clarifying the molecular characteristics and temporal changes of pyroptosis in NARDS is of great importance for its prevention and treatment [16].

In recent years, single‐cell RNA sequencing (scRNA‐seq) technology has been widely applied for the analysis of biological mechanisms [17]. Sequencing and analyzing the transcriptome of individual cells in the microenvironment of NARDS can not only reveal the heterogeneity of inflammatory cells but also effectively distinguish the characteristics of various cell subpopulations and lesion molecules in the neuroinflammatory microenvironment and propose effective treatment strategies [18]. Accordingly, we analyzed the main cell subsets that mediate the inflammatory storm in patients with NARDS using single‐cell combined transcriptome sequencing and found that lung macrophages played a key regulatory role [19]. Pulmonary macrophages comprise three types: bronchial macrophages, alveolar macrophages (AMs), and interstitial lung macrophages, among which AM account for >90%. During NARDS, AM exhibits strong heterogeneity and plasticity [20, 21]. Influenced by different tissue factors and microenvironments in the lungs, they can polarize into different phenotypes and release cytokines with regulatory effects. In the early stages of NARDS, AM transform into M1 macrophages, which secrete proinflammatory factors such as interleukin (IL) ‐1, tumor necrosis factor (TNF) α, IL‐6, IL‐8, IL‐12, and inducible nitric oxide synthase [22]. These inflammatory factors act on the macrophages themselves, activating more M1 macrophages, whereas these cytokines interact with epithelial cells, lymphocytes, and neutrophils, promoting the progression of inflammatory responses in the lungs. In addition, TNF can upregulate tissue factors, thereby promoting platelet aggregation and microthrombosis formation, as well as coagulation and hyaline membrane formation in the alveoli. Meanwhile, M2 macrophages secrete anti‐inflammatory factors, such as IL receptor antagonists (IL‐1ra), IL‐10, and arginase 1 (Arg1), which can neutralize the inflammatory response in the lungs [23]. The anti‐inflammatory factors include some factors terminate their functions by neutralizing or blocking inflammatory factors (IL‐1ra) [24], whereas others increase the density of IL‐4 receptors on the surface of macrophages, enhance the sensitivity of macrophages to IL‐4 and IL‐13, and facilitate their polarization into M2 macrophages [25]. During the development of NARDS, pyroptosis of alveolar M2 macrophages causes lung tissue repair dysfunction and an imbalance in the M1/M2 ratio, promoting inflammatory progression [26, 27]. Therefore, intervention strategies targeting pyroptosis in the AM have become a significant direction for the treatment of NARDS injuries.

**Figure 1:**
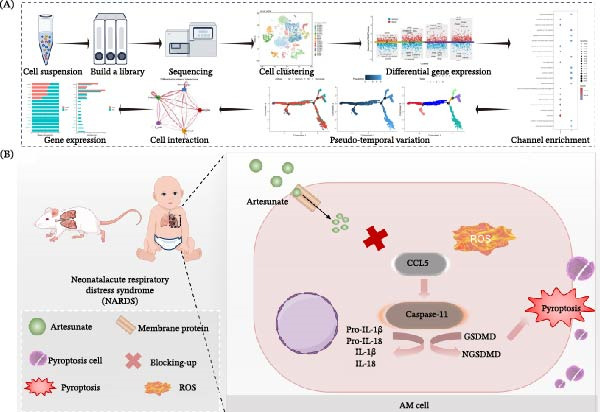
Schematic diagram of scientific hypotheses: pathogenesis and treatment strategies of Neonatal Respiratory Distress Syndrome. (A) Schematic diagram of the single‐cell analysis process for NARDS. (B) Molecular mechanism pathways of artemether in the treatment of NARDS.

Recent studies have shown that artesunate can exert multiple biological effects, including anti‐apoptotic and regulatory effects on lung tissue inflammation. Studies also suggest that artesunate has anti‐inflammatory and restorative effects and is a potential pyroptotic inflammatory protective agent [28]. It reduces neuroinflammation‐induced apoptosis by activating the ERK 1/2/CREB/bcl 2 signaling pathway. Furthermore, artemisinin could inhibit apoptosis, oxidative stress, and inflammatory molecular indicators [29, 30]. Currently, no clear evidence indicates that artesunate can effectively regulate pyroptosis in AM cells and improve NARDS [31]. Therefore, investigating the protective anti‐inflammatory effects of artesunate in NARDS in the context of pyroptosis may provide a novel mechanistic perspective (Figure 1).

## 2. Materials and Methods

### 2.1. Single‐Cell Sequencing and Bioinformatic Analysis

#### 2.1.1. Data Acquisition and Preprocessing of Sequencing Data

Single‐cell sequencing data related to NARDS were obtained from the Gene Expression Omnibus (GSE223793) database. This dataset consists of one experimental group and two control groups. Data preprocessing was performed using 10× Genomics Cell Ranger software (version 6.1.2). First, the raw sequencing data were subjected to strict quality control, resulting in approximately 15,436 cells. After quality control, 14,206 high‐quality cells were retained for subsequent analyses. The specific screening criteria were as follows: cells with <200 detected genes and those with mitochondrial gene expression ratios exceeding 20% were removed, and genes expressed in fewer than three cells were filtered out. Subsequently, the clean read sequences were mapped to the reference genome using the STAR alignment algorithm to generate the aligned BAM files. After alignment, the Cell Ranger counting function was used to identify cell barcodes, count unique molecular identifiers, and quantify gene expression, generating a final gene‐cell expression matrix.

#### 2.1.2. Dimensionality Reduction and Clustering Analysis of Single‐Cell Data

Normalization, identification of highly variable genes, and dimensionality reduction via PCA were conducted using Seurat 4.0, followed by cell clustering using the k‐nearest neighbor algorithm and the Leiden method. Cell subpopulations were annotated according to established marker genes to elucidate variations in the proportional abundance of macrophage subsets.

#### 2.1.3. Differentially Expressed Genes (DEGs) and Functional Enrichment Analysis

The Wilcoxon rank‐sum test was employed to identify DEGs based on a threshold of |log_2_ fold change (FC)| ≥ 1 and the adjusted *p*‐value. Volcano plots were generated to visualize the distribution of DEGs, distinguishing upregulated genes in red and downregulated genes in blue. Gene Ontology (GO) and Kyoto Encyclopedia of Genes and Genomes (KEGG) pathway analyses were conducted using the clusterProfiler 4.0 R package, with statistical significance defined as *p* < 0.05. Gene set enrichment analysis (GSEA) was performed using GSEA 4.1.0 software on hallmark gene sets (MSigDB), KEGG gene sets, and macrophage metabolism‐related signature gene sets, with significance thresholds set at FDR < 0.25 and NES ≥ 1.5. Core‐enriched genes were extracted to generate enrichment plots and heat maps, thereby elucidating the key metabolic pathways activated within the macrophage subpopulations.

#### 2.1.4. Analysis of Ligand–Receptor Interactions via CellChat

Intercellular communication was inferred by leveraging the ligand–receptor–transcription factor axis dataset derived from KEGG pathways. Based on prior knowledge of these interactions, the analysis integrated ligand and receptor expression profiles with the activity of specific downstream transcription factors to elucidate the signaling networks.

#### 2.1.5. Developmental Trajectory Analysis of Key Cell Subtypes

DEGs within the macrophage population were selected for trajectory inference. The cellular developmental trajectory was reconstructed using a learning graph function in the Monocle3 framework. A root node, designated as the starting cell, was defined to compute the pseudo‐time values for individual cells. Consequently, visualizing the pseudo‐time distribution, analyzing dynamic gene expression along the pseudo‐time axis, and identifying branch points elucidated the continuous state transitions of chondrocytes, validating the reliability of the trajectory.

### 2.2. Network Pharmacology and Molecular Docking to Investigate Artesunate Treating NARDS

#### 2.2.1. Screening of Artesunate Compound and Disease‐Related Targets

The standard chemical structure (2D/3D) and physicochemical properties of artesunate were obtained from PubChem (https://pubchem.ncbi.nlm.nih.gov/) to ensure structural integrity and accuracy. Structural preprocessing was performed using ChemDraw 20.0 to eliminate redundant atoms and bonds and optimize the molecular conformation for subsequent target prediction analysis. Disease‐associated targets were identified by interrogating the GeneCards, compound–target–disease (CTD), and Open Targets databases using the keyword ”neonatal respiratory distress syndrome.” Gene symbols were standardized, and duplicates were removed to establish a consolidated dataset of potential disease targets. The overlap of targets across different databases was visualized using Venn diagrams. The identified compound targets were intersected with the retrieved disease targets using the R statistical environment to isolate common targets relevant to the artesunate‐mediated treatment of NARDS for downstream analysis. Venn and UpSet plots illustrating the intersection of targets across databases were generated using the VennDiagram (v1.7.3) and UpSetR (v1.4.0) packages in R (v4.2.1).

#### 2.2.2. Construction and Topological Analysis of the Artesunate CTD Network

To delineate the interactions between artesunate and its target proteins, a CTD network was established using Cytoscape (version 3.9.1). Within this graphical representation, the nodes were defined as the active compound and its corresponding targets, whereas the edges illustrated the interactions among them. The topological architecture of the network was investigated using the NetworkAnalyzer plug‐in. This analysis facilitated the calculation of key network metrics, with the node degree being the primary parameter for discerning the relative importance of each node.

#### 2.2.3. Construction of the Protein–Protein Interaction (PPI) Network

A PPI network was generated using the STRING database to elucidate interactions among the screened intersection targets. The search was confined to *Homo sapiens*, employing a confidence score cutoff of ≥0.5 for the included interactions. The resulting data (TSV/PNG) were imported into Cytoscape, where the network was pruned by removing all isolated nodes. Subsequently, a topological interrogation of the network was performed. The pivotal targets or hubs were identified either by evaluating and sorting various topological metrics or by applying the maximal cluster centrality algorithm integrated in the CytoHubba plugin.

#### 2.2.4. GO and KEGG Pathway Enrichment Analyses

GO functional enrichment and KEGG pathway enrichment analyses were performed on relevant targets using the R package clusterProfiler (v4.6.2). For GO analysis, a significance threshold of *p* < 0.05 was applied, and the top 15 significant biological terms were visualized as bar charts. Similarly, KEGG pathway enrichment utilized a significance threshold of *p* < 0.05, with the top 30 significantly enriched signaling pathways visualized using bubble plots.

#### 2.2.5. Construction of the “Artesunate Compound–Target Pathway–Disease” Network

To investigate the underlying therapeutic mechanisms of artesunate, a global “compound–target pathway–disease” network was constructed by integrating the top‐ranked core pathways with their corresponding key targets and active constituents. This visualization highlights the synergistic interactions between key active constituents and therapeutic targets within critical signaling pathways. Molecular docking was performed using the AutoDock Vina software.

#### 2.2.6. Molecular Docking Simulations

This study used molecular docking to probe the binding affinities between pivotal active compounds and their core protein targets. The requisite three‐dimensional (3D) chemical structures of the ligands were obtained from PubChem. The crystal structures of the target proteins were downloaded from the Protein Data Bank (PDB; https://www.rcsb.org/). Prior to docking, protein structures were prepared using PyMOL (v2.6.0), a process involving the elimination of solvent molecules. AutoDock Tools (v1.5.7) was used to incorporate polar hydrogen atoms and compute Gasteiger charges for both the protein and ligand structures, which were saved in the requisite PDBQT format. A specific grid box was parameterized to encompass the binding site of interest. Finally, the resulting docking poses, which depicted the predicted binding modes, were visualized and interpreted using PyMOL.

### 2.3. In Vitro Experiments

#### 2.3.1. Assessment of Cell Viability and Biocompatibility

Bone marrow‐derived macrophages (BMDMs) were isolated from the bone marrow of SPF‐grade male C57BL/6 mice. The mice were intraperitoneally injected with an excessive 0.3% pentobarbital sodium solution at a dose of 0.15 mL/10 g. After the reflex in the foot disappeared, all animals were euthanized by cervical dislocation in accordance with the American Veterinary Medical Association (AVMA) animal euthanasia guidelines, and the carcasses were disinfected with 75% alcohol (Animal Ethics Approval Number: GDY2202454). The harvested cells were cultured in a mixed medium composed of α‐minimum essential medium and Dulbecco’s modified Eagle’s medium, which was supplemented with 10% fetal bovine serum and 1% penicillin–streptomycin mixture. Subsequently, the cell cultures were incubated at 37°C in a humidified incubator in a 5% CO_2_ atmosphere.

#### 2.3.2. Cell Pyroptosis Model Induction

To take logarithmic growth phase BMDMs, cell suspension was adjusted to a density of 2.5 × 10^5^/mL using complete culture medium and inoculated into a 6‐well plate at a volume of 1 mL per well. It was then placed in a 37°C, 5% CO_2_ incubator for overnight culture for 24 h to allow the cells to adhere. After adhesion, the cell density was approximately 70%. For experimental grouping, cells in the culture plate were divided into two groups with three biological replicates per group. The groups included a blank control group, a group with only an equal volume of complete culture medium, and a lipopolysaccharide (LPS) + ATP combined induction group. LPS was added at a final concentration of 3 μg/mL for 16 h, followed by the addition of ATP at a final concentration of 5 mM for 2 h to induce pyroptosis.

#### 2.3.3. Artesunate Intervention Experiment on Cell Pyroptosis

To evaluate the effect of artesunate on pyroptosis, cells in the logarithmic growth phase were inoculated into 6‐well plates or Petri dishes. After the cells reached a 70% confluence, subsequent processing was performed. Artesunate was prepared as a stock solution in dimethyl sulfoxide and diluted with a complete medium to a working concentration of 5 μmol/L. Cells with induced pyroptosis were further incubated for 24 h. After the treatment, the cells and culture supernatants were collected for subsequent detection of lactate dehydrogenase (LDH) release, caspase‐11 activity, expression changes of pyroptosis‐related proteins, and genes such as IL‐1β.

#### 2.3.4. Polymerase Chain Reaction (PCR)

The method described by Yang et al. [32] was followed. Quantitative PCR (qPCR) reactions were assembled in a 25 μL volume. The mixture consisted of 12.5 μL of 2× Taq PCR master mix, 0.5 μL of each gene‐specific primer (10 μM, refer to Table S1 for sequences), 1 μL of the DNA template, and nuclease‐free water to volume. Thermal cycling was conducted on a real‐time qPCR system with the following protocol: initial DNA denaturation at 95°C for 5 min, followed by 40 cycles of 95°C for 30 s (denaturation), a 30‐s annealing step, and 72°C for 30 s (extension). A final elongation at 72°C for 10 min was performed, after which the samples were stored at 4°C. The experimental design incorporated technical triplicates for each sample to ensure data reliability, along with a negative control (without a template) and a verified positive control.

#### 2.3.5. Immunofluorescence Staining

The method described by Yang et al. [32] was used. BMDMs were seeded onto confocal dishes and maintained at 37°C under 5% CO_2_ for 24 h until reaching a 60%–70% confluence. After removing the culture medium, the cells were gently rinsed three times with prewarmed phosphate‐buffered saline (PBS) and fixed using 4% paraformaldehyde for 15 min. The cells were then permeabilized with 0.3% Triton X‐100 and washed three times with PBS. To minimize nonspecific background staining, a blocking step was performed with 5% bovine serum albumin (BSA) at 37°C for 30 min. Following the removal of the blocking solution, the cells were treated with specific primary antibodies (Table S2) overnight at 4°C. The next day, the samples were incubated at room temperature for 30 min and washed three times with PBS. Subsequently, the cells were exposed to the corresponding secondary antibodies for 1 h at room temperature in the dark. After incubation, the unbound secondary antibodies were removed by three 5‐min washes with PBS. Finally, cell nuclei were stained with Hoechst 33342 and imaged using a ZEISS LSM 980 confocal laser scanning microscope (Carl Zeiss, Germany).

#### 2.3.6. Western Blot Analysis

For protein extraction, cells were washed with pre‐chilled PBS and lysed using a chilled buffer formulation supplemented with a cocktail of protease and phosphatase inhibitors to preserve protein integrity and phosphorylation. Complete cell disruption was achieved by a combination of chemical lysis and sonication on ice. The lysate was clarified by centrifugation at 4°C, and the resulting supernatant, representing the total protein fraction, was transferred to fresh tubes and stored at –20°C until use. Protein concentrations were determined using a BCA protein assay with BSA as the standard. For immunoblotting, protein samples were combined with the loading buffer and denatured by boiling at 100°C for 10 min. Equal protein amounts (2 μg/μL) from each sample were separated by sodium dodecyl sulfate‐polyacrylamide gel electrophoresis and then transferred onto a polyvinylidene fluoride membrane. To minimize nonspecific background signals, the membrane was blocked with a rapid blocking buffer. It was then probed with specific primary antibodies (Table S2) at 4°C overnight. Following incubation with the corresponding secondary antibodies, antigen–antibody complexes were detected using an ECL substrate. Chemiluminescent signals were documented using a GelView 6000 Pro system, and the acquired digital images were analyzed using ImageJ to quantify the density of each band. The method described by Yang et al. [32] was followed.

### 2.4. Statistical Analysis

This study utilized GraphPad Prism 10 (GraphPad Software, USA) and SPSS version 23 (IBM Corp., Armonk, NY, USA) for all statistical analyses. Continuous data are reported as mean ± standard deviation, unless otherwise specified. Intergroup comparisons of multigroup data were conducted using one‐way ANOVA (followed by Bonferroni’s test) for parametric data or the Kruskal–Wallis test for nonparametric data. A *p*‐value of <0.05 was considered statistically significant, with specific thresholds denoted as  ^∗^ *p* < 0.05,  ^∗∗^ *p* < 0.01, and  ^∗∗∗^ *p* < 0.001.

## 3. Results and Discussion

### 3.1. Single‐Cell Sequencing Reveals the Characteristics and Changes of Subtypes of Cell Populations in NARDS

NARDS poses a serious threat to the life and health of newborns. However, the cellular and molecular targets that influence the prognosis of children with NARDS remain unclear. To examine the cellular characteristics of NARDS, NARDS RNA‐Seq and transcriptome datasets were selected for comprehensive analysis. This analysis first identified the main cell types present in the injured lung tissue, followed by a detailed analysis of their subpopulations. Common cell populations were defined based on established cell markers, such as neutrophils, macrophages, fibroblasts, T cells, lymphocytes, natural killer cells, alveolar epithelial cells, and precursor B cells (Figure 2A–D). Meanwhile, a heat map analysis of the specific genes in each cell subpopulation was conducted to observe their expression profiles in lung tissue samples (Figure 2E,F). The bar chart shows the upregulation of DEGs in the control and NARDS groups. Genes, including *IGLC3*, *IGHA1*, *JCHAIN*, *S100A3*, and *HS3STSA1*, showed higher expression levels in macrophages. The expression of *LYPD2*, *PTGDS*, *APOBEC3B*, *MALL*, and *GATA6* was upregulated in monocytes and that of *CCL17*, *CCL22*, *IDO1*, *FCER1A*, and *CCL19* in M1 macrophages. The expression of genes, such as *CDC45*, *SYT4*, TSLP, *BLK*, and *SLC4A10*, increased in M2 macrophages (Figure 2G). The changes in the proportions of these cell subpopulations and alterations in their specific gene expression profiles suggest that the occurrence and development of NARDS may be closely related to the remodeling of the immune‐inflammatory microenvironment. First, the significant upregulation of multiple inflammation‐related chemokines CCL17, CCL22, and CCL19 and immune regulatory molecules IDO1 and FCER1A in macrophages and their different polarization states (M1/M2) indicate that the activation of the innate immune system and the imbalance of immune regulation in NRDS may be important factors that drive lung tissue damage. Second, the upregulation of genes, such as *APOBEC3B* and *GATA6*, in monocytes/lymphocytes suggests that the adaptive immune response and cell differentiation process may be involved in the pathogenesis of NARDS, further exacerbating the inflammatory response or affecting tissue repair. Additionally, the abnormal expression of immune protein‐related genes, such as *IGLC3*, *IGHA1*, and *JCHAIN*, in macrophages indicates that the enhanced local humoral immune response may be related to impaired alveolar barrier function and inflammatory cascade reactions. Moreover, the upregulation of some genes related to cell proliferation and signal transduction, such as *CDC45*, *SYT4*, and *TSLP*, in M2 macrophages may reflect the tissue repair and remodeling processes initiated by the body in the context of inflammatory injury. However, this process may have a regulatory imbalance, thereby leading to pathological changes. In summary, this study reveals the dynamic changes and molecular characteristics of various immune cell subpopulations in the lung tissue of patients with NARDS at the single‐cell level, suggesting that the activation of the inflammatory response, imbalance of immune regulation, and abnormal tissue repair may jointly participate in the occurrence and development of NARDS. These findings provide a new theoretical basis for further screening of potential therapeutic targets and for improving the prognosis of children with NARDS.

Figure 2The changes in subtype characteristics and differential expression of cell populations in the experimental group and the control group in NARDS. (A–D) Clustering of cell subgroups. (E, F) Heatmaps of genes specifically expressed by each cell subgroup. (G) Heatmaps of genes with increased and decreased expression in each cell subgroup.

**Figure:**
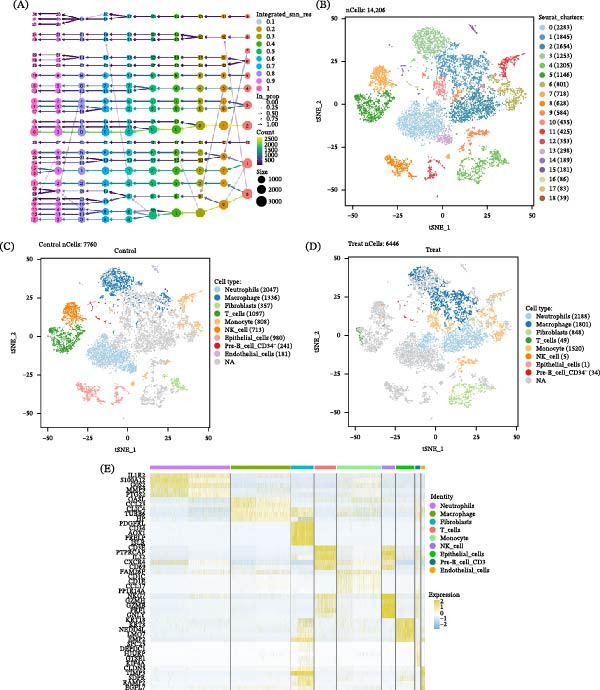

**Figure:**
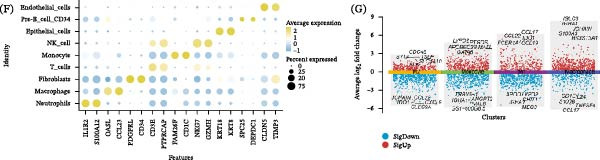

### 3.2. Identification of Macrophage Subsets and Their Differences Among Different Samples

The functional homeostasis of cells that form lung tissues is crucial for NARDS progression. Among them, monocytes/macrophages are the dominant immune cells in the lung tissue. In the early acute exudation stage of NARDS, most resident AMs immediately transform into the M1 proinflammatory phenotype. AMs are the first line of defense against pathogenic microorganisms and damage to lung tissues. After eliminating virulence factors, the resident and recruited macrophages transform from the M1 phenotype to the anti‐inflammatory M2 phenotype. M2 macrophages limit the levels of proinflammatory factors, which help produce anti‐inflammatory cytokines and phagocytose apoptotic neutrophils, ultimately halting the proinflammatory response. Accordingly, we conducted further hierarchical cluster analysis of NARDS‐specific cell subpopulations (Figure 3A–C), which were defined by the molecular markers (cell markers) *MRC1*, *CD163*, *MERTK*, *CD38*, *CD86*, *CSF1R*, *CD14*, and *APOE*, including M1 macrophages, M2 macrophages, and mononuclear macrophages, and compared them with normal controls. The proportions of M2 and M1 macrophages and mononuclear macrophages in the NARDS group significantly increased (Figure 3D). In parallel, through the enrichment of GO and KEGG pathways, the characteristic genes of immune cell subsets were further labeled, and these genes were found to be closely related to genes involved in the inflammatory response, respiratory regulation, immune inflammation, repair of cellular vascular injury, and programmed cell death in T17 cells (Figure 3E,F). M2 macrophages are anti‐inflammatory cells crucial in inflammatory repair. Changes in the proportion of inflammatory cells suggest that M2 macrophages are key in tissue injury healing.

Figure 3M2 macrophage subsets play an important role in the repair of NARDS. In the NARDS group, M2 macrophages enhanced phagocytic clearance, inflammatory regulation, and injury repair, and were involved in pyroptosis of lung tissue cells. (A–C) Clustering analysis of cell subgroups. (D) Genes specifically expressed by M1 type macrophages, M2 type macrophages, and monocyte‐macrophages. (E, F) GO and KEGG pathways were used to annotate the characteristic genes of immune cell subgroups.

**Figure:**
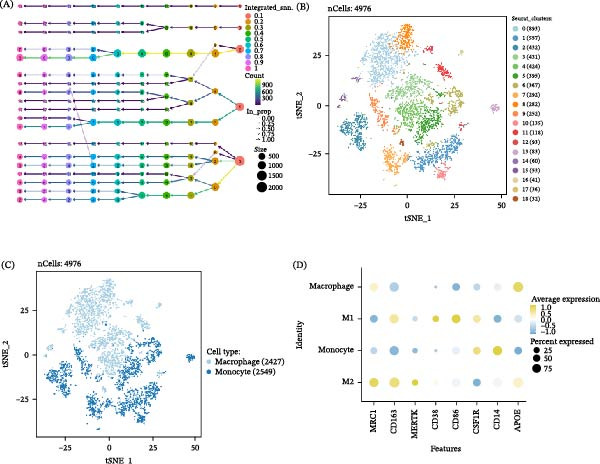

**Figure:**
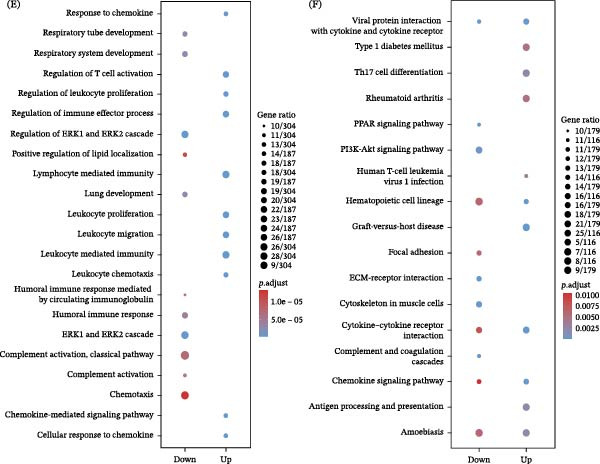

### 3.3. M2 Macrophages Enhance Phagocytic Clearance, Inflammatory Regulation, Tissue Repair, and Are Involved in the Occurrence of Pyroptosis in the NARDS Group

**Figure 4:**
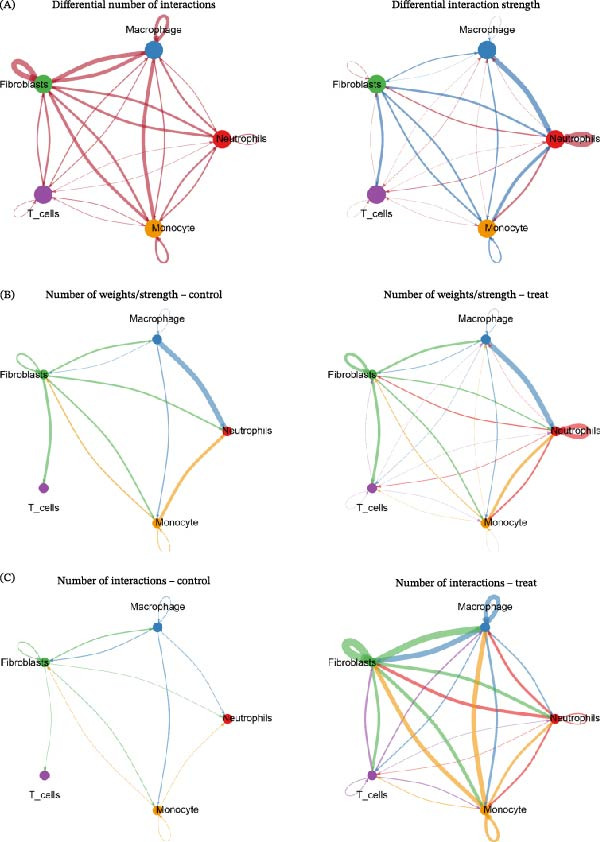
The intensity and quantity of the interaction between M2 macrophages and other cells in the lung tissue in the NARDS group. (A) The interaction intensity between cells in the control group and the experimental group. (B, C) The interaction intensity and quantity between macrophages in the control group and the experimental group and various cell subpopulations.

Pyroptosis is a novel form of cell death closely related to the inflammatory response. It is mediated by inflammasomes, cysteine proteases, and members of the GSDM protein family, causing the formation of pores in the cell membrane and the release of inflammatory mediators, thereby inducing inflammatory responses. Pyroptosis is common during the course of NARDS and closely related to the occurrence of inflammation after lung tissue injury in ARDS. To verify the role of M2 macrophages in the repair of NARDS inflammatory damage, intercellular interaction relationships, and regulatory mechanisms, the Cellchat package (version number) was initially used for cell interaction analysis, which revealed that the number and intensity of interactions in the experimental group (NARDS group) were significantly higher than those in the control group (Figure 4A). The analysis showed that the interactions between macrophages and other cells were more common (Figures 4B,C and 5A). In addition, the gene pathway enrichment heatmap showed that in the macrophage subpopulation, compared with other cell subpopulations, the expression of inflammatory response regulation pathways, pyroptosis, positive regulation of inflammatory stimulation, and Toll‐like receptor pathways was significant (Figure 5B). In the inflammatory and pyroptosis pathways, the gene expression levels of *CCL*, *ANGPTL*, *VEGF*, *EDN*, *RANKL*, *CD137*, *CD70*, *PTN*, *QX40*, and *PARs* in the NARDS group were significantly higher than those in the control group, whereas the expression levels of *MK*, EGF, *IL-1*, *PROS*, and *CXCL*, among others, decreased (Figure 5C). Further analysis revealed that the alterations in gene expression in the NARDS group were significantly associated with pyroptosis, phagocytic clearance, inflammatory regulation, and injury repair. *CCL* may be a specific gene that affects cell pyroptosis, thereby altering injury repair in NARDS tissues.

Figure 5CCL was specifically highly expressed in the NARDS group. (A) Heatmap of interactions between macrophages and other cells. (B) Among the macrophage subpopulations, the gene pathway enrichment heatmap indicates that the expression of inflammatory response regulation pathways, programmed necrosis, positive regulation of inflammatory stimulation, and Toll‐like receptor pathways has significantly increased. (C) Differentially expressed genes in the NARDS group.

**Figure:**
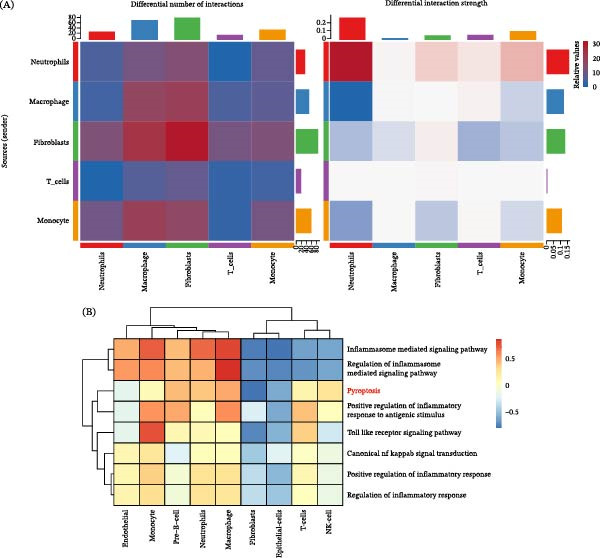

**Figure:**
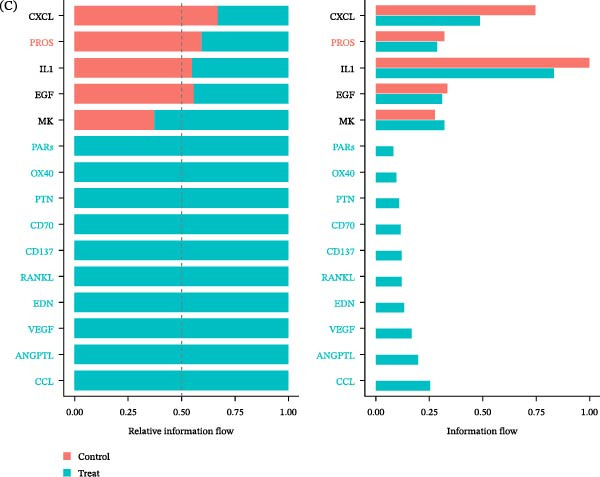

### 3.4. Trajectory Analysis Shows the Differentiation Trajectories of Macrophages

**Figure 6:**
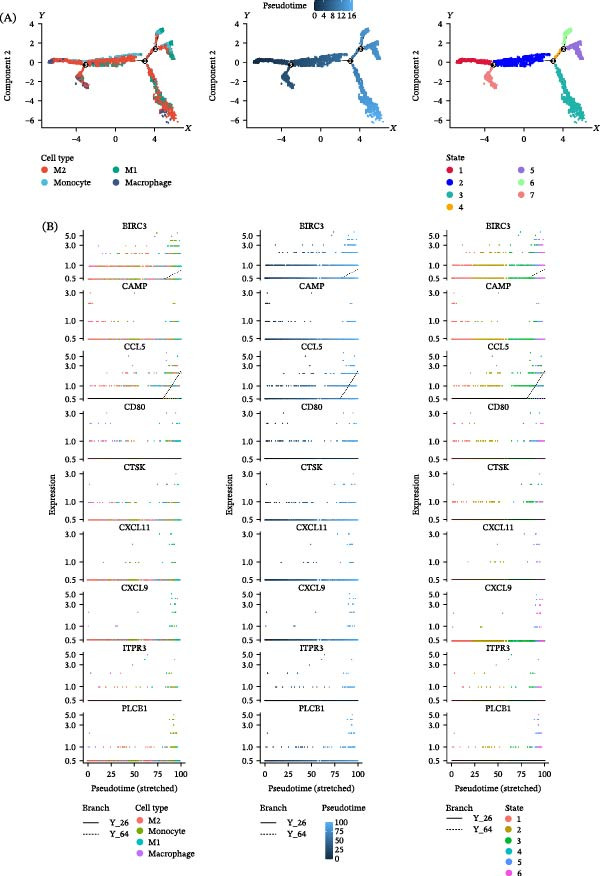
Trajectory analysis shows the differentiation trajectory of M2 macrophages. (A) Trajectory analysis of macrophages. (B) Significantly regulates the expression of CCL5 in genes that control M1 macrophage polarization.

To investigate the differentiation process and mechanisms of macrophages in tissue repair, a quasi‐temporal analysis of macrophage subsets was conducted using monocle2 (version number). Cells were classified into three states (Figure 6A), with M2 differentiating from M1 and mononuclear macrophages. Genes that regulate the polarization of M1–M2, such as *BIRC3*, *CAMP*, *CCL5*, *CD80*, *CTSK*, *CXCL11*, *CXCL9*, *ITPR3*, and *PLCB1*, were highly expressed in M1 (Figure 6B). The aforementioned studies revealed a tendency for M1–M2 polarization in the NARDS group. Moreover, *CCL5* may be closely associated with a protective mechanism against tissue inflammatory injury.

### 3.5. In Vitro Experiments Verified that the Specific High Expression of CCL Affects the Occurrence of Pyroptosis in M2 Macrophages

**Figure 7:**
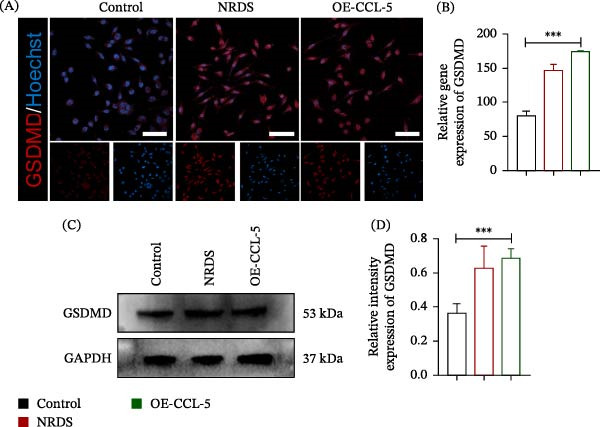
In vitro experiments were conducted to verify the effect of CCL5 expression on pyroptosis. (A) Fluorescence expression of GSDMD protein in the experimental group, NARDS group and CCL5 overexpression group (*n* = 3). (B) Fluorescence quantitative analysis of GSDMD protein (*n* = 3). (C) The WB expression of GSDMD protein in the experimental group, NARDS group, and CCL5 overexpression group (*n* = 3). (D) Quantitative analysis of GSDMD protein by WB (*n* = 3). One‐way analysis of variance using the Bonferroni multiple comparison test ( ^∗^ *p* < 0.05,  ^∗∗^ *p* < 0.01,  ^∗∗∗^ *p* < 0.001).

To verify the expression of CCL5 in M2 macrophage subsets, immunofluorescence and western blotting were performed to determine changes in the protein expression of GSDMD among the disease, normal group and CCL5 overexpression group. As shown in Figure 7A,B, significant differences were found in the expression of the GSDMD protein among the disease, normal, and CCL5 overexpression groups. In addition, western blotting was performed to observe the expression changes of the GSDMD protein in each group (Figure 7C,D), which was consistent with the immunofluorescence results. These results indicate that the abnormal expression of CCL5 in the NARDS group may be the main factor influencing inflammation in NARDS. *CCL5* is a family of genes encoding closeness centrality (CC)‐like chemokines and belongs to the largest subclass of the chemokine superfamily. These genes play key roles in immune responses, inflammatory reactions, and disease development by regulating the migration, activation, and functions of immune cells. In view of this, we speculated that targeted drugs or molecular inhibitors regulating CCL5 expression may improve the occurrence of cell death mediated by inflammation in NARDS.

### 3.6. Network Pharmacology Reveals That Artesunate Has a Potential Therapeutic Effect on the Pyroptosis Regulatory Gene CCL5 of M2 Macrophages

To investigate therapeutic interventions targeting the M2 macrophage pyroptosis gene CCL5, GeneCards (score ≥30), CTD (score ≥10), and Open Targets (score ≥0.01) databases were interrogated. Following threshold‐based filtering and gene symbol standardization, 1394 disease‐associated targets were identified. Potential disease targets associated with CCL5 intersected with 269 targets corresponding to the active constituents of artesunate. This mapping revealed 57 common targets that potentially mediate the therapeutic effects of artesunate on NARDS (Figure 8A). A CTD network was constructed (Figure 8B) comprising 59 nodes and 114 edges, with the edges delineating specific compound–target interactions. The interaction with 57 targets uncovered the multicomponent, multitarget pharmacological mechanism characteristic of artesunate, wherein distinct active constituents modulate shared targets and individual constituents exert pleiotropic effects. The 57 intersecting targets implicated in the NARDS treatment were imported into the STRING database to generate a PPI network (Figure 8C). The topological properties were analyzed using a network analyzer. Node size and color intensity were stratified by degree values, and the top 30 hub nodes were identified based on degree, betweenness centrality (BC), and CC rankings. The key therapeutic targets identified within the core cluster included CCL, ALB, CASP3, EGFR, ESR1, TNF, SRC, PPARG, MMP9, and MAPK1. Pathway enrichment analysis of the 57 intersection targets was conducted using the R package clusterProfiler, and the top 30 enriched pathways were visualized in a bubble plot (Figure 8D). Enrichment analysis revealed a predominance of TNF, HIF‐1, and platinum drug resistance pathways, suggesting that these pathways exert synergistic therapeutic efficacy. Consequently, after integrating the degree ranking of the active constituents (Figure 8E) with literature validation, molecular docking was performed between artesunate and the top 10 hub targets (CCL, ALB, CASP3, EGFR, ESR1, TNF, SRC, PPARG, MMP9, and MAPK1), as illustrated in Figure 8F,G. The molecular docking simulation results show that artemether has a strong binding affinity with key targets such as CCL, PPARG, and EGFR with binding energies of –9.07, –8.61, and –8.26 kcal·mol, respectively. CCL forms hydrogen bonds with the TRY‐90, GLU‐64, and ASN‐7 residues, confirming its potential as a targeted therapeutic drug for regulating M2 macrophage pyroptosis in NARDS.

Figure 8Network pharmacology elucidates the targeted recognition of CCL5 molecules by artesunate in NARDS‐associated macrophages. (A) Venn diagram illustrating the intersecting targets between artesunate and NARDS. (B) Construction of the artesunate‐target–disease interaction network. (C) Protein–protein Interaction (PPI) network analysis. (D) Sankey‐bubble plot visualizing the top‐ranked targets and signaling pathways. (E) Integrated artesunate‐target‐pathway‐disease network. (F) Heatmap displaying the molecular docking binding energies between top active components and primary targets. (G) Molecular docking visualization showing the interaction between active ingredients and CCL5 target molecules.

**Figure:**
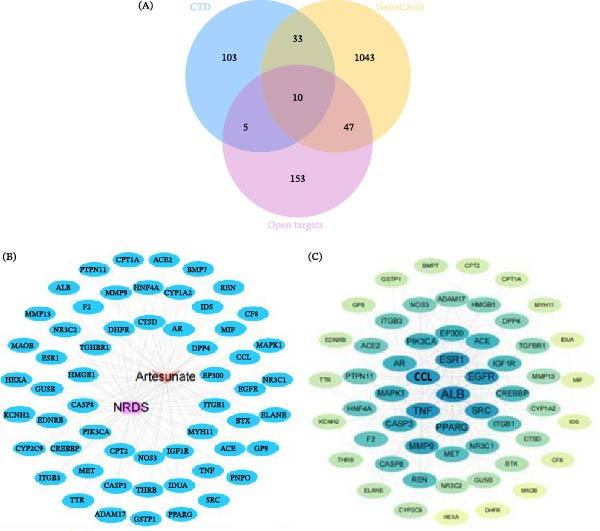

**Figure:**
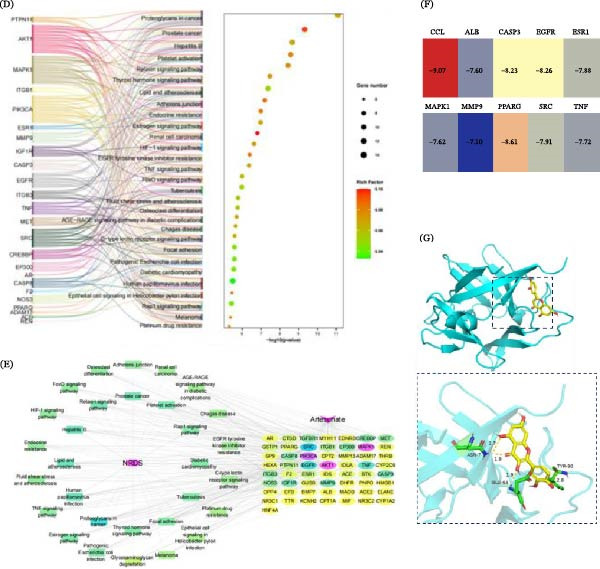

### 3.7. Artesunate Regulates CCL5 Expression to Inhibit Pyroptosis in M2 Macrophages and Thereby Improve Inflammatory Damage in NARDS

**Figure 9:**
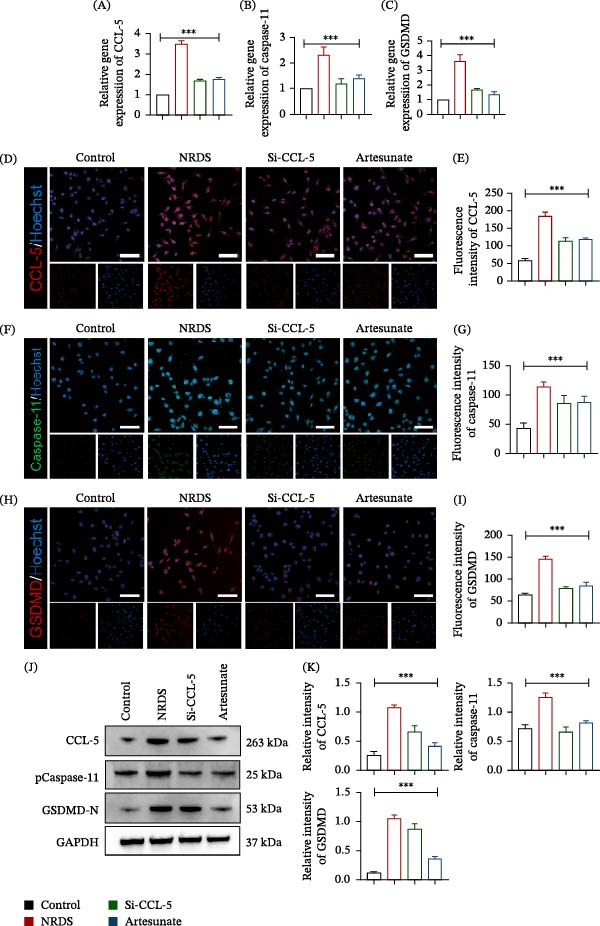
The expression of CCL5 and pyroptosis after artesunate treatment. PCR of CCL5, caspase‐11, and GSDMD gene expression in the (A–C) experimental group, NARDS group, Si‐CCL5, and artesunate group (*n* = 3). Fluorescence expression and quantitative analysis of CCL5, caspase‐11, and GSDMD proteins in the (D–I) experimental group, NARDS group, Si‐CCL5, and artesunate group (*n* = 3). (J, K) WB expression and quantitative analysis of CCL5, caspase‐11, and GSDMD proteins in the experimental group, NARDS group, Si‐CCL5, and artesunate group (*n* = 3). One‐way analysis of variance using the Bonferroni multiple comparison test ( ^∗^ *p* < 0.05,  ^∗∗^ *p* < 0.01,  ^∗∗∗^ *p* < 0.001).

**Figure 10:**
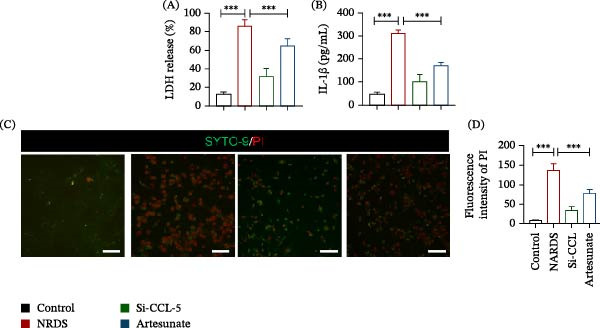
The expression of CCL5 and pyroptosis after artesunate treatment. (A) Lactate dehydrogenase (LDH) release assay (*n* = 3). (B) IL‐1β secretion analysis by ELISA (*n* = 3). (C,D) Propidium iodide (PI) staining for cell death (*n* = 3). One‐way analysis of variance using the Bonferroni multiple comparison test ( ^∗^ *p* < 0.05,  ^∗∗^ *p* < 0.01,  ^∗∗∗^ *p* < 0.001).

Artesunate, a derivative of artemisinin, has antimalarial, anti‐inflammatory, and immune‐regulating activities and the potential to inhibit pyroptosis. First, artesunate reduces the caspase‐1‐dependent pyroptosis pathway by inhibiting reactive oxygen species (ROS) or by scavenging free radicals, thereby blocking the ROS‐NLRP3 inflammasome activation axis. Second, it may interfere with the activation of nonclassical pyroptosis pathways (such as caspase‐4/11), inhibit the cleavage of GSDMD and formation of cell membrane pores induced by LPS. In addition, artesunate can indirectly inhibit the pyroptosis‐related inflammatory cascade reaction by reducing the release of proinflammatory factors (IL‐1β and IL‐18). Accordingly, the inhibition of pyroptosis and improvement of inflammatory symptoms in NARDS were observed after treatment with artesunate. By culturing M2 macrophages and applying LPS oligomers for intervention, the results revealed that compared with the CON group, the expression level of the *CCL5* gene in the NARDS group was significantly increased, whereas in the si‐CCL5 and artesunate groups, the expression level of *CCL5* was downregulated compared with that in the disease group (Figure 9A–C). The results of the western blotting and immunofluorescence assays were consistent with those of the PCR. Furthermore, the expression levels of the pyroptosis‐related pathway proteins GSDMD and caspase‐11 were detected. In the NARDS group with high CCL5 protein expression, caspase‐11 was significantly increased. That is, compared with the si‐CCL5 and artesunate groups, the expression level of GSDMD was significantly lower than that in the NARDS group, and the differences were statistically significant (Figure 9D–K). The LDH release experiment results showed that compared with the control group, NARDS treatment significantly increased the LDH level in the cell supernatant, suggesting damage to the cell membrane integrity; on this basis, CCL5 knockdown significantly reduced LDH release, and artesunate also significantly decreased the LDH level (Figure 10A). Consistently, the ELISA detection results indicated significantly increased secretion of IL‐1β in the cell supernatant of the NARDS group, and CCL5 knockdown and artesunate significantly inhibited the release of IL‐1β, effectively controlling the inflammatory response (Figure 10B). Furthermore, PI staining revealed that the proportion of PI‐positive cells in the NARDS group was significantly increased, presenting a stronger red fluorescence signal, whereas CCL5 knockdown and artesunate treatment significantly reduced the number of PI‐positive cells (Figure 10C,D). These results collectively indicate that under NARDS conditions, cell membrane permeability increases and the release of inflammatory factors is enhanced, whereas CCL5 can exacerbate this process and artesunate has a significant inhibitory effect.

This study has some limitations. Data analysis was conducted using public databases, and the results were only verified at the cellular level without testing in vivo animal models or clinical tissue specimens. The clinical‐related prognosis and inflammatory levels were not fully demonstrated. In subsequent studies, we will explore the correlation between the expression levels of MARDS molecular targets in the body and diseases.

## 4. Conclusions

NARDS is a critical respiratory condition that seriously threatens a newborn’s life. It progresses rapidly and is associated with a high fatality rate [33]. The inflammatory response is an important pathophysiological process in NARDS. Previous studies have suggested that pyroptosis mainly occurs in classical inflammatory cells, M1 macrophages, and is mediated through the NLRP3 inflammasome and caspase‐1 pathways [34]. However, recent studies have also indicated that the nonclassical caspase‐11‐dependent pathway is crucial in acute lung injury. In the highly inflammatory and damaged microenvironment of NARDS, M2 macrophages may experience a functional imbalance because of continuous stimulation, and the chemokines they secrete promote the inflammatory cascade and amplify pyroptosis, thereby hindering tissue repair [35]. Interventions targeting the pyroptotic pathway may provide new directions for treating NARDS [36].

In this study, scRNA‐seq was employed to examine the molecular mechanisms of pyroptosis in the lung tissue microenvironment of patients with NARDS. Our analysis identifies the inflammatory chemokine CCL5 in M2 macrophages as a key player in regulating pyroptosis and hindering tissue repair [37]. Mechanically, CCL5 regulates the noncanonical pyroptosis pathway by interacting with inflammasome components, thereby amplifying the cleavage of caspase‐11‐dependent GSDMD and the release of IL‐1β [38]. Therefore, this study screened and experimentally verified the role of artesunate in inhibiting CCL5 expression and reducing pyroptosis in M2 macrophages using network pharmacology. Given that it has been approved for clinical use and has a clear safety foundation, its application in NARDS has a high potential for clinical translation. Artesunate significantly alleviates pyroptosis in M2 macrophages, reduces the area of lung injury, and decreases lung inflammation. Our research results not only suggest that CCL5 is a new regulator of pyroptosis in macrophages but also propose artesunate as a promising NARDS therapeutic agent targeting pyroptosis and inflammation. This study provides a mechanistic basis for the development of CCL5‐based intervention measures and highlights the dual anti‐inflammatory and restorative effects of artesunate in the treatment of NARDS.

## Author Contributions

**Hai-Ran Ma:** conceptualization, methodology, formal analysis, validation, investigation, writing – original draft. **Yan-Mei Xie:** writing – review and editing, methodology, validation, formal analysis. **Yu-Xuan Zhang:** methodology. **Chong-He Cui:** validation. **Jing Liu:** conceptualization, methodology, writing – review and editing, supervision, data curation, project administration.

## Funding

No funding was received for this study.

## Disclosure

All authors have contributed to the article and approved the submitted version.

## Ethics Statement

All animal experiments were carried out in the Animal Experimental Center of Huizhou Central People’s Hospital. All operations were trained, and the experimental protocol was reviewed and approved by the Ethics Committee of Huizhou Central People’s Hospital.

## Conflicts of Interest

The authors declare no conflicts of interest.

## Supporting Information

Additional supporting information can be found online in the Supporting Information section.

## Supporting information


**Supporting Information** Table S1. Primer sequences of each gene. Table S2. Related information about primary antibodies.

## Data Availability

The data are available upon request from the authors.
