# Supplementary material for: Artesunate Alleviates Inflammatory Injury in Neonatal Acute Respiratory Distress Syndrome Through CCL5‐Mediated Pyroptosis of M2 Macrophage Subsets
Source: J Immunol Res. 2026 Aug 5;2026:7437447. doi: 10.1155/jimr/7437447 (PMC13443700; doi:10.1155/jimr/7437447)
Supplement: Supplementary file 1 [file JIMR-2026-7437447-s001.docx]

# Supplementary Description

| Table S1 - Primer sequences of each gene. | | |
| --- | --- | --- |
| Target | Forward | Reverse |
| CCL | CCTGCTGGTCTTCCTCATTG | AGGTGGTGGATTTGCTGATG |
| GSDMD | ATGGCCTCTGCTGCTGCTG | TCAGACTTGGGGCTGGTCTC |
| Caspase-11 | GACAGCAACGTGCTGGACTT | TGTGGGTAGAGGGTGGTTTC |

| Table S2 - Related information about primary antibodies | | | | | |  |
| --- | --- | --- | --- | --- | --- | --- |
| Catalog  Number | Product  Name | Species  Reactivity | Dilution  (cell/tissue) | Molecular  weight | Source | |
| 60004-1-Ig | GAPDH | Rabbit | 1:1000 | 37 kDa | Proteintech, China | |
| 12000-1-AP | CCL5 | Rabbit | 1:1000 | 8 kDa | Proteintech, China | |
| 20770-1-AP | GSDMD | Rabbit | 1:1000 | 53 kDa | Proteintech, China | |
| #2227 | Caspase-11 | Rabbit | 1:1000 | 25 kDa | Cell Signaling Technology | |
